# Measurements of the hydrodynamic pressure on a surfboard fin during surfing

**DOI:** 10.1038/s41598-025-94834-0

**Published:** 2025-03-25

**Authors:** Stefan Kniesburges, Nina Punger, Bogac Tur, Michael Zöllner, Marc in het Panhuis, Michael Döllinger

**Affiliations:** 1 Department of Otorhinolaryngology, University Hospital Erlangen, Friedrich-Alexander University Erlangen-Nürnberg, 91054 Erlangen, Germany; 2 Institute of Information Systems, Hof University, 95213 Münchberg, Germany; 3 Surf Flex Lab, Faculty of Engineering and Information Sciences, University of Wollongong, Wollongong, NSW 2500 Australia; 4 School of Science, University of Wollongong, Wollongong, NSW 2500 Australia

**Keywords:** Bone quality and biomechanics, Fluid dynamics, Techniques and instrumentation, Engineering

## Abstract

The popularity of surfing has increased during the last 20 years with the growing number of river waves and artificial wave pools. For these different surfing conditions, hydrodynamic characteristics of boards and fins and their optimization become interesting for industry and science to analyze the biomechanics and physiology during surfing. In this work, a measuring system was developed assembled of four small pressure sensors included in a 3D-printed fin within a 2-fin configuration. The measurements were controlled by an acquisition board mounted into a surfboard. The system was initially tested in a water tank and exhibited a high accuracy of measured pressure. Afterwards, a surfer surfed the instrumented surfboard on a river wave and performed three cycles of surfing from one side of the wave channel to the other. The results showed a pressure difference between both sides of the instrumented fin that produces periodical lift forces directed away from the surfboard. Thereby, the maximum lift force was produced during the surfer’s motion from one side of the channel side to the other. It is assumed to increase the stability of the surfer’s back foot in combination with the right fin producing a lift force in opposite direction.

## Introduction

The popularity of surfing has been increased over the last 20 years. Beside famous surfing spots in Australia, America and Asia, more and more river surfing facilities have been established, e.g. Germany’s renowned surf spots such as the *Munich Eisbach* and the *Nürnberger Fuchslochwelle*. Additional wave pools as the *Kelly Slater Surfing Ranch* in Leemore/California or the *URBNSURF wave pool* in Melbourne/Australia provide many possibilities to go surfing for everyone living far off the coasts. Whereas surfing has a long tradition in the historical surfing regions, such as, e.g. Hawaii and Australia with well-organized competitive sports environments, even countries like Germany have started to organize national competitions, e.g. the German Championship in Rapid Surfing in 2019 in Cologne. As a consequence of the rise of popularity and the resulting increase of professional competitive structures, surfing has been included in the program of the Olympic games since 2016.

With the large increase in popularity, McArthur et al.^1^ reported an exponential increase in individuals surfing on river or pool waves in non-coastal regions and highlighted the lack of sufficient data related to surf-specific injuries. They identified the three most common injury mechanisms in coastal surfing as follows: collision/impact with one’s own surfboard (38.5%), injuries during maneuvers or wave passage (20.3%), and impact with the seafloor (18.4%) after falling off the surfboard. The affected body parts were primarily head, face, and neck (33.8%) and the lower extremities (33%). To reduce the risk of sports injuries and improve performance, surfers have to possess specific biomechanic characteristics, such as a specific morphology of skeletal muscles^2^. However, to identify these specific anatomical and physiological characteristics, a physiological surfer’s model has to be developed that provides the insight in the acting hydrodynamic forces and the surfer’s biomechanics. To establish such a model, detailed information about the complex interaction between surfer, surfboard, and wave have to be analyzed in computer simulations and real surfing situations. However, especially the experimental acquisition of the hydrodynamic forces and the surfboard’s and surfer’s dynamics is difficult to acquire.

In this context, the scientific literature lacks of studies about influence of different surfboard designs and technical equipment for analyzing surfing dynamics. In fact, the technical development of surf equipment (surfboards, surfboard fins, surfer’s clothing, etc.), is primarily driven by industrial companies. Additionally, traditional manufacturers (individuals known as *shapers*) have been playing a key role in developing geometric shapes of surfboards and fins based on subjective observations from surfers to enhance surfing performance^3^.

Furthermore, scientific studies on the hydrodynamic properties are extremely rare. Romanin et al.^4^ listed only 17 scientific contributions in a review article in 2021 (13 journal publications and 4 conference proceedings). Among these, 11 studies focused on the hydrodynamic properties of surfboards (3×)^5–7^ and fins (7×)^3,8–12^, while the remaining 7 studies analyzed specific features of surfers’ wetsuits^13–19^.

Due to the small number of available scientific studies, the reliability of previous studies especially in hydrodynamics has to be interpreted carefully for the following reasons. Computational models have been highly simplified and included mostly only the fins without the surfboard. Furthermore, only static surfing situations have been investigated with mostly steady flow conditions. The entire surfboard and fins on a free surface have not been addressed yet. Moreover, the most critical point is that detailed experimental studies performed using test channels and/or waves (for validating the numerical simulations of the interaction between the surfboard’s hydrodynamics, structural mechanics and the surfer’s motion) have not yet been realized.

In fact, experimental studies of surfing mostly focus on acquiring the trajectories, velocities and acceleration data during surf sessions based on GPS in combination with motion sensors^11,20–22^. All these studies pursue the goal to measure surfing performance to quantify the performance in professional surfing competitions and to analyze the bio-mechanical work and loads for optimization of training concepts^23–25^. Based on these techniques in combination with surfers’ self evaluations, Forsyth et al.^26^ made a first attempt to acquire the performance of innovative fin designs and found a correlation between the differences of surfers’ perception for different fin types with the GPS trajectories and inertial motion data.

However, to investigate the complete process of surfing, the hydrodynamics at the bottom side of the surfboard, the surfer’s posturing on the surfboard and the interaction between both have to be measured simultaneously during surfing sessions. For the last two tasks, first studies have been published based on video analysis of the body’s posture in combination with artificial intelligence^27,28^ and pressure sensors between the surfer’s feet and the surfboard^29^.

To measure the hydrodynamics at the bottom side of the surfboard, the large challenge is to develop a robust measuring system that can be embedded in the surfboard that comprises the sensors, the acquisition and the storing of the measured data as well as the power supply for the all components. In this regard, Krzyzanowski and in het Panhuis^30^ introduced an embedded measuring system with deformation sensors integrated in a fin for detecting its flexing deformation during surfing on waves. They found flexing ratios of the fins of up to 10% at surfing speeds of up to $6\,$m/s. Beside this indirect measure of the fin’s pressure load, no direct hydrodynamic quantities have been measured yet.

Thus, the primary aim of this study is to introduce a pressure measuring system integrated in a 3D printed surfboard fin prototype based on the geometry of a commercial left fin that enables the evaluation of hydrodynamic pressure conditions on fins during surfing on a river wave (*Fuchslochwelle* in Nürnberg/Germany). Beside the validation of hydrodynamic computer simulations that are potentially included in a complete computer model of surfing, the proposed pressure measurement system will be useful and an important tool in the development of innovative fin designs that can enhance surfing performance, e.g. the humpback whale inspired fins by Shormann and in het Panhuis^11^.

## Results

### Laboratory diving test

The relative hydrostatic pressure can be calculated as function of the depth of water ($L_{wd}$) using the following Eq. 1^31^:

1 $$\begin{aligned} P_{hs}=\rho \, g\,L_{wd} \end{aligned}$$

with $\rho$ being the density of water and *g* the gravitational acceleration.

The mean hydrostatic pressure $P_{hs}^*$ measured by the two sensors in the watertight container, the standard deviation $\sigma _P$ and the relative error *e* for each depth of water are displayed in Table 1. It shows a linear increase from 0 Pa to 2.45 kPa for a water depth down to 25 cm that was the maximum depth during the experiments. The mean relative error for the two sensors were 0.61% and 0.63%, respectively, with a range between 0.02 and 3.9% for different water depths.

**Table 1:** Water tank measurements: depth of water $L_{wd}$, hydrostatic pressure $P_{hs}$ according to Eq. 1, mean measured pressure $P_{hs}^*$ with its standard deviation $\sigma _P$ and the relative error *e* of the measurements compared with the analytically computed pressure $P_{hs}$ (Eq. 1 at constant depths of water).

$L_{wd}$/cm	$P_{hs}$/Pa	$P_{hs}^*\pm \sigma _P$/Pa	Error *e*/%
2.5	245.25	$250.5\pm 10.70$	3.9
5.0	490.5	$493.1\pm 14.03$	2.4
7.5	735.75	$725.2\pm 7.85$	1.4
10.0	981	$978.7\pm 11.33$	1.0
12.5	1226.25	$1222.4\pm 10.18$	0.8
15.0	1471.5	$1463.9\pm 10.66$	0.8
17.5	1716.75	$1714.6\pm 9.54$	0.5
20.0	1962	$1959.2\pm 4.00$	0.2
22.5	2207.25	$2206.3\pm 6.32$	0.2
25.0	2452.5	$2454.5\pm 5.61$	0.2

### Pressure measurement during surfing maneuvers

After the laboratory tests in the water tank, the fin with four sensors instrumented was mounted onto a surfboard. The surfboard was then used by an experienced river surfer who performed several turns on the river wave.

**Fig. 1:**
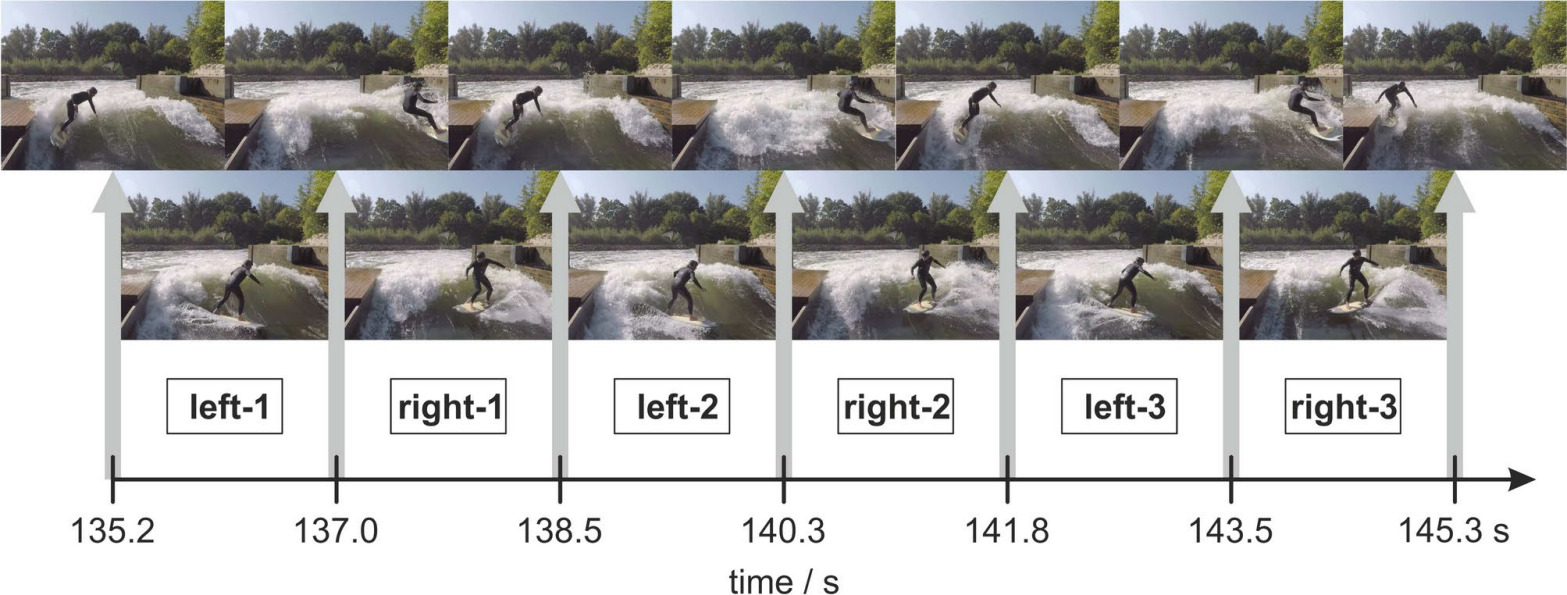
Series of pictures recorded by the GoPro camera showing the surfer performing three cycles (1–3) of a left-right ride between both sides of the wave channel. The time points of the position of either side are shown on the time axis below the pictures with vertical arrows showing the corresponding pictures.

Figure 1 shows images of specific time points during the maneuvers extracted from the video footage. In total, three complete runs, each starting on the left, traveling to the right and back to left have been performed. The periods of traveling are indicated by left or right with the number of the respective run, e.g. in the period left 1, the surfer traveled from the right to the left channel border.

In the upper row of Fig. 1, the surfboard was aligned in parallel to the main flow direction during the turning maneuver at either side of the channel. It marks the channel border and the change of directions between the travel periods towards the right or left channel border. The exact time points of these pictures are indicated by the gray arrows.

In the lower row, the surfer is in the mid position of the channel traveling from one channel side to the other which corresponds to the temporal mid position between the according pictures in the upper row.

**Fig. 2:**
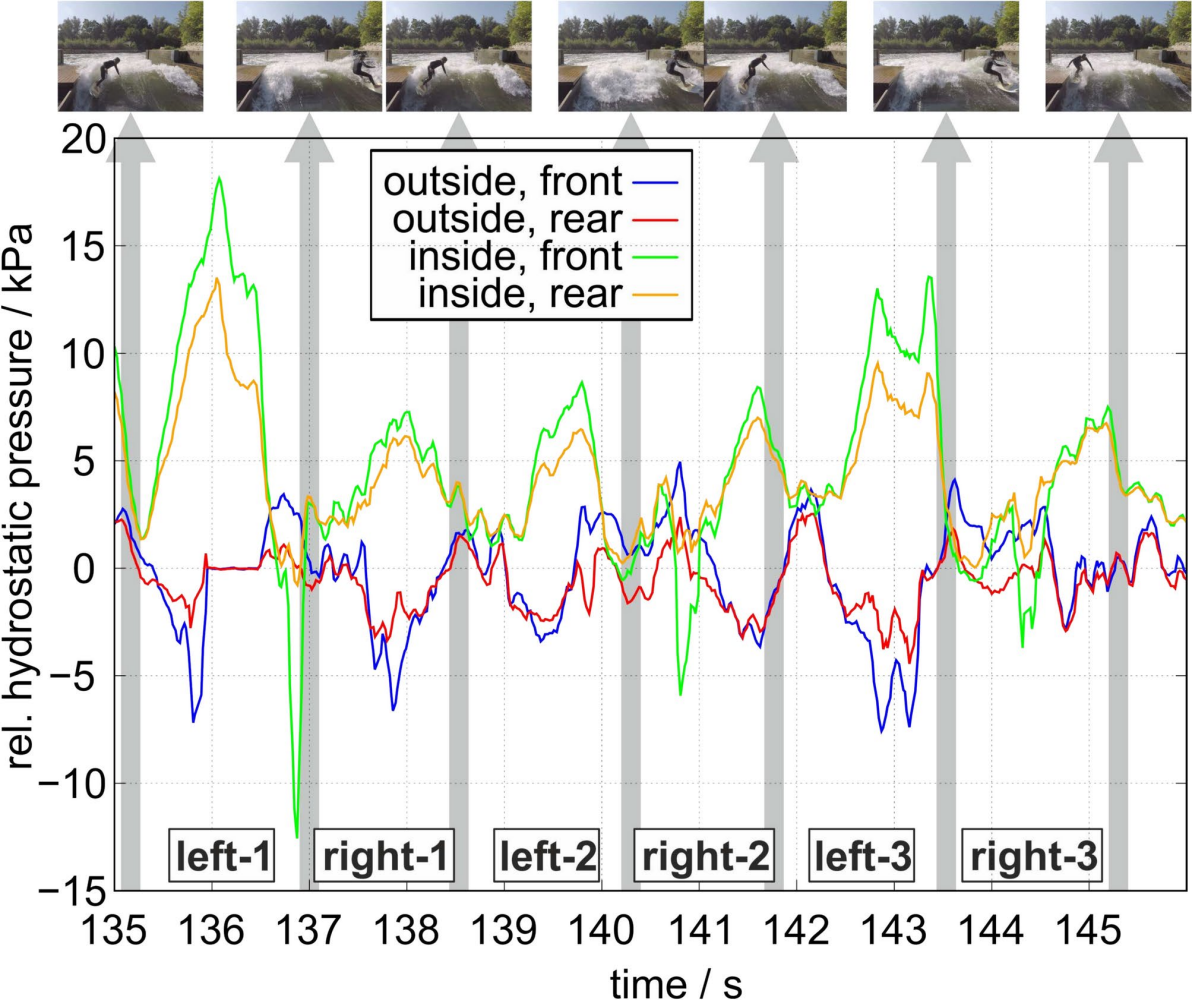
Waveform of the relative hydrostatic pressure measured by the four sensors in the left fin for the period shown in Fig. 1. The sensors are indicated by their location on the outside or inside surface of the fin in frontal or rear position according to Fig. 6. The time points when the surfer was either side of the channel are indicated by the gray bars with the pictures of the surfer on top. The traveling periods are named accordingly with left-1 to right-3.

Synchronously to the image recordings of the surfer, the pressure distribution on the left fin is depicted in Fig. 2 measured by the four pressure sensors mounted on either side of the fin as described above. The gray bars indicate the time point of parallel surfboard alignment with flow shown in Fig. 1.

The pressure distribution measured by all four sensors shows similar and systematic patterns. During all traveling periods of the surfboard from one side of the channel to the other, a pressure difference between the outside and the inside surfaces of the left fin occurs for each oppositely mounted sensor pair. The maximum value of pressure difference ranges approximately between 14 and 20 kPa. Thereby, these maximums are located in the traveling periods slightly shifted towards the end of each traveling period with the sign of the pressure difference staying the same regardless the direction which the surfer traveled (to the left or right). Furthermore, the value of the pressure difference was almost quite similar except the traveling for periods left-1 and left-3 which were much larger mainly for the opposite sensor pair at the front position of the fin.

**Fig. 3:**
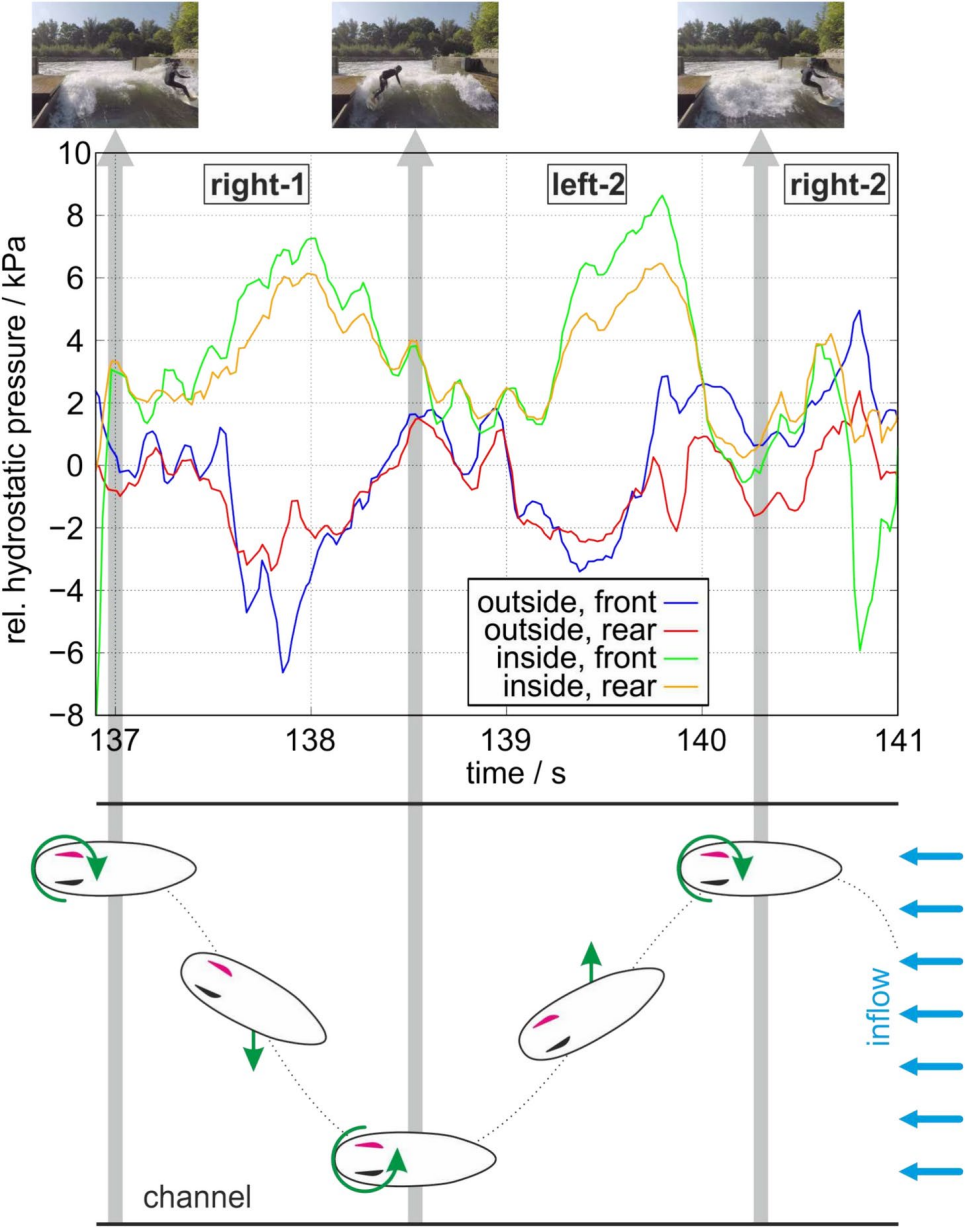
Top: Zoom representation of the pressure waveform in Fig. 2 for the right-1 and left-2 traveling periods indicated in the diagram. According to Fig. 2, the time points when the surfer was either side of the channel are indicated by the gray bars with the pictures of the surfer on top. Bottom: Schematic orientation of the surfboard and the motion characteristic with the inflow of the wave coming from the right.

For analyzing this characteristic behavior of the pressure distribution, Fig. 3 shows the close-up of the traveling periods right-1 and left-2 in the top part with a schematic representation of the surfboard orientation in the bottom part of the figure with the left and equipped fin colored in red. Thereby, each surfboard in its specific orientation corresponds to the pressure distribution at this time point shown in the top part of Fig. 3. The figure shows that the maximum pressure difference occurs when the surfer was half way of his travel from one side to the other where he possessed the highest velocity. The pressure difference between outside and inside surface is very similar during right-1 and left-2 with a maximum value of approximately $10\,$kPa.

## Discussion

The experimental data represent the first hydrodynamic parameters measured during real surfing maneuvers. The large challenge was to include the pressure sensors in the fins and to wire them with the data acquisition board included water-tightly in the surfboard’s body. A very similar measuring system was also developed by Krzyznowski and in het Panhuis^30^ who measured the mechanical tension and flex in surfboard fins during real surfing maneuvers.

The equipped fin produced a classical lift force similar to the airfoils of planes as the fin possess a wing profile shown in Fig. 6 with the curved outside surface directed in outward and the flat inside surface in inward direction. Owing to the mounting position and orientation of the left equipped fin, the resulting pressure force is directed to the outside direction which was also reported in several computational studies of three^12,32^ and four-fin configurations^3,9^.

**Fig. 4:**
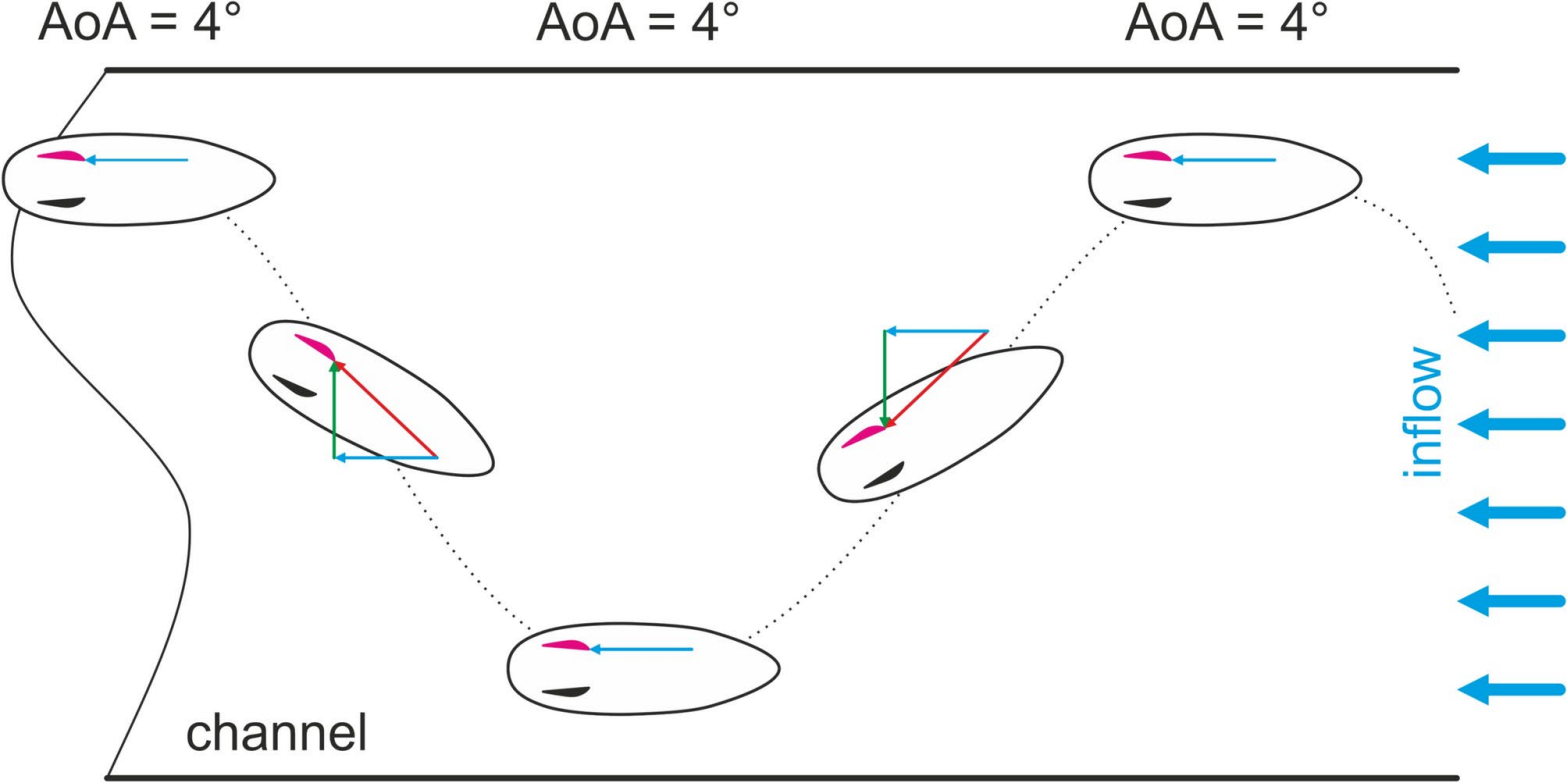
Schematic orientation of the surfboard with the inflow velocities at different phases during a right to left ride. The arrows indicate the water inflow from the wave in blue, superposed by the inflow due to the surfers motion traveling from one side to another (green) and the resulting inflow drawn in red.

Most interestingly, the pressure difference between the inside and outside surface of the left fin and, thus, the lift force became maximum during the traveling periods and was directed in the same direction regardless whether the surfer traveled to the right or left. It is assumed that the negative pressure at the curved outside of the left fin is maintained for both travel direction as the fin is always appropriately aligned with the inflow additionally supported by the $4^{\circ }$ toe-in angle during the left traveling motion as displayed in Fig. 6. Thereby, the water velocity of the flow is superposed with the surfer’s traveling velocity producing a velocity vector with higher absolute velocity producing the maximum pressure difference which is aligned with the wing profile of the fin as shown in red in Fig. 4. This increase in lift force confirms the findings in most of the computational studies which reported an increase in lift coefficient with increasing angle of attack^9,12,32^. As indicated in Fig. 4, the angle of attack (AoA) is assumed to be much lower than $5^{\circ }$ which falls in the ranges of the simulation-based studies.

As the right fin is symmetrically mounted in the surfboard, it can be assumed that the right fin also produces a similar pressure distribution with a negative pressure at the curved outside and a high positive pressure at the inside fin surface. This also produces a lift force that is directed in the opposite direction to the force at the left fin.

The measured relative pressures at the fin went down to $-7\,$kPa on the curved outside and up to $17\,$kPa on the flat inside surface of the left fin. The maximum pressure difference between outside and inside surface amounts approximately $20\,$kPa. Assuming a homogenous pressure distribution on the entire fin surface with $0.015\,\text {m}^2$, the resulting maximum lift force can be estimated to approx. $f_l=300\,$N.

This lift force is similar to those reported in computational studies in^3,12,32^ for AoAs up to $5^{\circ }$ as estimated above. In Falk et al.^12^, the entire 3-fin configuration produced a lift force of $f_l=96\,$N for an $\text {AoA}=5^{\circ }$ and an inflow velocity of $5\,$m/s. Hereby, the large reduction in lift force might be the result of the oppositely directed lift force at the opposite fin when considering both the left and the right fin for computing the lift force. In the study reported by Crameri et al.^32^, the authors reported lift forces of $160\,$N, $185\,$N and $200\,$N for three fins with increasing fin surfaces between $0.020\,\text {m}^2$ and $0.0236\,\text {m}^2$ applying an inflow velocity of $7\,$m/s. The difference might be caused by the overrating of the maximum lift force found experimentally in this study due to the assumption of homogenous pressure distribution over the entire surface of the fin. Sakellariou et al.^8^ reported lift forces in the range of $f_l=50\,$N for $10\,$m/s inflow and $AoA=10^{\circ }$. They optimized the fin’s shape to maximize the lift/drag ratio considering a fin with a surface of $0.01\,\text {m}^2$. In Shoremann et al.^10^, the authors simulated a cutback turn maneuver and reported mean resultant forces of approx. $300\,$N acquired at 101 data points on the fin surface during the turn. This seems to coincide very well with the data measured in this work, however, the cutback turn is a very fast maneuver in contrast to the slow turn carried out in this study whereas the fast traveling periods during the ride has not been considered in^10^. It can be concluded at this point, that only computed values of pressure and lift forces at fins obtained in CFD simulations are available for comparison. Nevertheless, the experimental data reported in this study match well with the range of computed lift forces. Differences can be attributed to different settings (inflow velocity, model types, different fin designs) and simulated maneuvers.

### Surfing performance: control, maneuverability, stability

In terms of surfing performance, large oppositely directed lift forces at the outer fins enhance resistance to turbulent flow fluctuations, thereby increasing the surfboard’s stability. This effect becomes larger for higher velocities of the surfboard reaching its maximum during the sideway travels on the river wave when the pressure difference and with it the lift forces become maximum as depicted in Fig. 3. Similar interpretations were concluded by Gudimetla et al.^9^ and Falk et al.^3,12^ who found increasing lift forces at the outside fins for increasing AoAs. The stabilization effect generated by opposing lift forces is concentrated at the rear of the surfboard, where the surfer’s back foot provides control and balance.

In typical maneuvers such as bottom turn, the surfer rotates the board through changes in weight distribution, i.e. leaning into the inside rail of the board, which is the side closest to the wave’s face, while simultaneously rotating their upper body and looking in the direction they want to go. This action (which “tilts” the board), combined with the pressure on the back foot, allows the fins to grip the water and propel the board upwards and around the wave’s curve. In contrast, the surfer’s front foot primarily acts as a balancing point, providing stability and guiding the surfboard’s direction. In other words, the front foot helps to steer while the back foot drives the maneuver corresponding to the Fig. 3.

Thus, it can be assumed that large, opposing lift forces significantly enhance maneuverability and control by providing adequate stability for the back foot, which serves as the rotation axis during rapid direction changes. However, the increased lift is accompanied by additional drag^3^, which can resist quick transitions and require greater effort from the surfer to initiate turns, particularly at high speeds.

Moreover, the degree of stability is directly influenced by the magnitude of the lift forces, making it a critical factor for maneuverability and control. From a surfer’s perspective, higher lift forces result in greater stability, which can become exceptionally strong and rigid. As a consequence, initiating direction changes requires significantly greater biomechanical effort due to increased resistance.

This effect becomes particularly pronounced in big-wave surfing (waves exceeding 6.2 m), where surfers must descend the steep wave faces at high speeds to avoid wipeouts, which can be extremely dangerous. At velocities ranging from 50 to $80\,$km/h, the lift forces generated by the board and fins become substantial, contributing to a high level of stability or even rigidity against turbulent flow fluctuations at the wave’s surface. However, this increased lift accompanied by greater drag resists quick transitions and makes rapid maneuvering more difficult.

The relationship between lift and drag presents a trade-off: while higher lift forces enhance stability, they also increase hydrodynamic resistance, which limits maneuverability. This is particularly evident in big-wave surfing, where high speeds amplify drag forces, causing the board to feel more resistant to directional changes. It is well known that big wave surfers need to initiate their bottom turns earlier and with a more shallower AoA to manage the wave’s power.

### Limitations

Although the measuring system worked very reliably and the acquired data delivered valid hydrodynamic data, the study has some limitations. The presented data just capture a short period of surfing with very simple and slowly performed turns executed by one male surfer on a well designed river wave. In this context, as shown above, the functionality of the pressure sensors and the validity of the corresponding pressure values have been checked in a water tank at static hydrostatic conditions. However, the pressure data were captured in a surfing situation at hydrodynamic conditions with a flow velocity of approx. $4\,$m/s. Small flow fluctuations originating from turbulence or vortex-shedding at the fins may not have been measured accurately owing to the reduced temporal resolution of the measuring system.

Those information would be important to evaluate the flow interaction between the different fins in multiple-fin configurations as Thruster and quad. As shown by Falk et al.^12^ in a 3-fin configuration, vortices shed from a outside fin might may excite the center fin to vibrations influencing the stability of the surfer in worst case. Another hardware-related limiting issue is the system’s applicability in commercial surfboards and fins of different brands and designs. Future development progress will improve the measuring system with respect to get easily mounted in commercial boards and fins at the lowest cost expense as possible. Having met these requirements, large case studies with male and female surfers at different skill levels and bio-mechanically individual body characteristics can be carried out helping to increase the understanding of surfing from physiological/bio-mechanical as well as hydrodynamical point of view.

## Conclusion

The hydrodynamics of surfboards have been previously analyzed in numerical simulations as measurements in real surfing situations are very challenging to perform and the transfer to the laboratory is quite limited. Therefore, a measuring system has been developed and introduced in this work that includes small pressure sensors in one fin of a real surfboard. The pressure data were measured and stored by an electronic data acquisition board that was water-tightly mounted within the surfboard and wired with the sensors.

After tests and the validation within a water tank, the equipped surfboard was ridden on a river wave and the surfer performed normal turns surfing back and forth from side of the channel to the other. The pressure distribution exhibits the typical airfoil behavior with a negative pressure and a positive pressure side as the fin possessed a typical wing-profile. The resulting lift force pointed in outside direction away from the surfboard.

To the best of our knowledge, the presented data are the first measured data of the hydrodynamic pressure acquired during real surfing maneuvers. They support the findings reported in computational studies qualitatively and quantitatively.

The introduced measuring system has significant potential for future applications in the scientific analysis of surfing dynamics, as well as in industrial design and product development. Its integration with additional sensors enables the measurement of key parameters such as overall velocity, transverse and rotational acceleration, surfboard orientation, surfer movement, and mechanical loads within the board and fins. These capabilities facilitate large-scale studies across various skill levels and demographics, providing deeper insights into the biomechanics of surfing.

Furthermore, the system allows for the generation of extensive datasets encompassing diverse wave conditions, including wave height and speed at different surf locations worldwide. When combined with global meteorological data and ocean swell forecasts, these datasets can contribute to the validation and refinement of wave prediction algorithms.

From a commercial perspective, real-time acquisition of quantitative surfing parameters enables data-driven optimization of surfboard and fin designs. By integrating subjective feedback from experienced surfers, this approach enhances the development process, translating perceptible differences in surfing characteristics into specific design modifications, such as surfboard flex behaviour, damping, fin shape, surface curvature, and rail geometry.

Finally, the collected data offers valuable insights for both professional athletes and recreational surfers by quantifying biomechanical loads on the body. Coupled with visual assessments of body posture, movement, and surfing trajectories, the hydrodynamic data provides a comprehensive framework for performance analysis and training optimization.

## Methods

### Pressure sensors and micro-controller

The identification of the correct pressure sensors is challenging due to extremely limited installation space in both the surfboard and fin as well as the measuring environment. Most commercially available fins for performance surfboards exhibited a maximum thickness of approximately $7\,$mm. The sensors and the installation conditions have to be waterproof. Furthermore, the maximum possible water depth the sensor has to work in was defined to be $2\,$m corresponding to a pressure of approx. $19.6\,$kPa relative to the atmospheric pressure. Based on these requirements, the absolute pressure sensor HSPPAD143C (Alps Alpine Electronic Inc., Tokio, Japan) has been chosen. The sensor includes a piezo-resistive bridge circuit on a silicon membrane. It is embedded in an application-specific integrated circuit for signal conditioning with temperature correction and exhibits an A/D converter and a digital I2C communication port (NXP Semiconductors N.V., Eindhoven, Netherlands). The sensor possesses a pressure range between $-700$ to $1000\,$hPa relative to the atmospheric pressure corresponding to a maximum water depth of $10\,$m.

The entire measuring system includes four pressure sensors connected via a TC9548A multiplexer (Texas Instruments Incorporated, Dallas/TX, USA) to an Arduino Nano 33 micro-controller (Arduino Srl, Monza, Italia). The measurement data are stored on a microSD card. The communication was performed via the I2S bus. The system is powered by two rechargeable lithium-polymer batteries providing $3.7\,$V and a combined capacity of $300\,$mAh. These components have been installed on a lead frame with the dimensions of $90\,$mm length and $70\,$mm width. The maximum sampling frequency of one sensor amounted to $f_s=200\,$Hz. The controller was fixed in a watertight box that was integrated in the surfboard similar to^30^.

The software program for the measurements was written with the open-source Arduino IDE software^33^ and transferred to the controller via a Micro-B USB interface installed on the controller. All components except of the pressure sensors were brazed on a conductor board which is named as data acquisition board in the following.

### Fin design and sensor integration

**Fig. 5:**
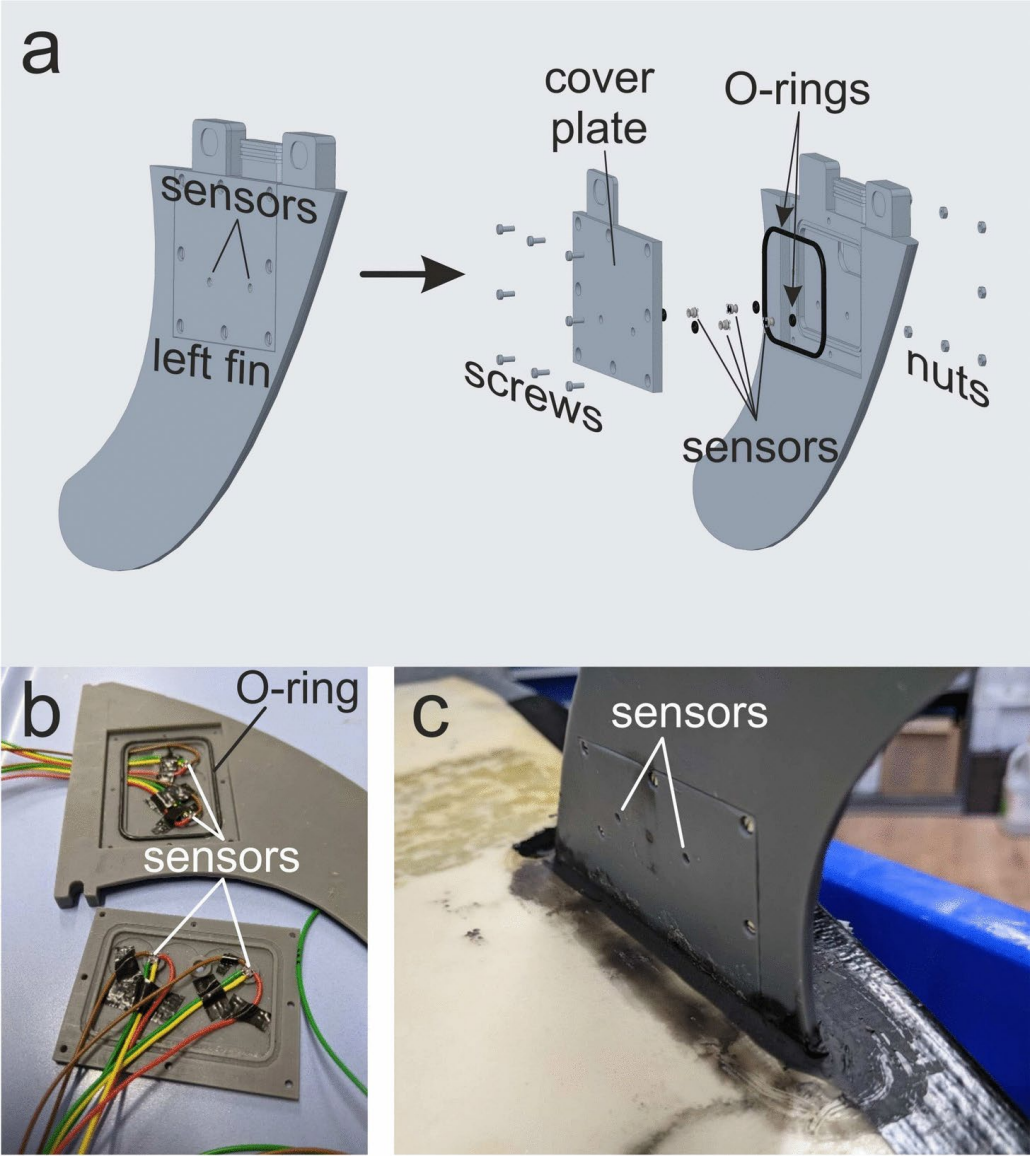
Images of the equipped left fin. (**a**) CAD model of the left fin of type FCS G5 Tri instrumented with four pressure sensors (explosion view) with FCS connector geometry. (**b**) Photo of the open left fin with the wires to connect to the data acquisition board with Futures connector geometry. (**c**) Photo of the left fin mounted in the surfboard.

The four pressure sensors were mounted in the left fin as shown in Fig. 5a. To include the space for the sensors, the fin geometry obtained from the GrapCAD Community (Stratasys Inc., USA)^34^ was adapted within the Creo Parametric CAD software (PTC Inc., USA)^35^ and 3D-printed by a Formlabs Form 2 stereo lithography (SL) printer (Formlabs GmbH, Germany) having a geometry similar to a fin of type FCS G5 Tri (FCS, Queensland, Australia). Two sensors were mounted within the main fin body and two sensors at opposite positions in the plate to measure the pressure at both sides of the fin. Each sensor was held in place by a small O-ring with a diameter $1.78\,$mm and a thickness $1.02\,$mm. The plate has the dimensions of $60\times 45\,\text {mm}^2$ and was mounted to the fin by eight M1.6x4 screws each with a length of $4\,$mm. It has to be noted here, that the geometry of the adapter of the fin was changed to the Futures type owing to a short-term change of the surfboard used.

To provide watertight conditions inside of the fin, a large O-ring with a diameter of $48\,$mm and a thickness of $1.5\,$mm was placed between plate and fin shown in Fig. 5b, c. Each sensor was cable-connected to the multiplexer with cables which were threaded through a small channel guided through the mounting opening in the surfboard into the watertight box to the data acquisition board. The channel from the fin to the waterproof box with data acquisition board was milled into the surfboard and made watertight with common sanitary silicone.

### Surfboard with data acquisition system

**Fig. 6:**
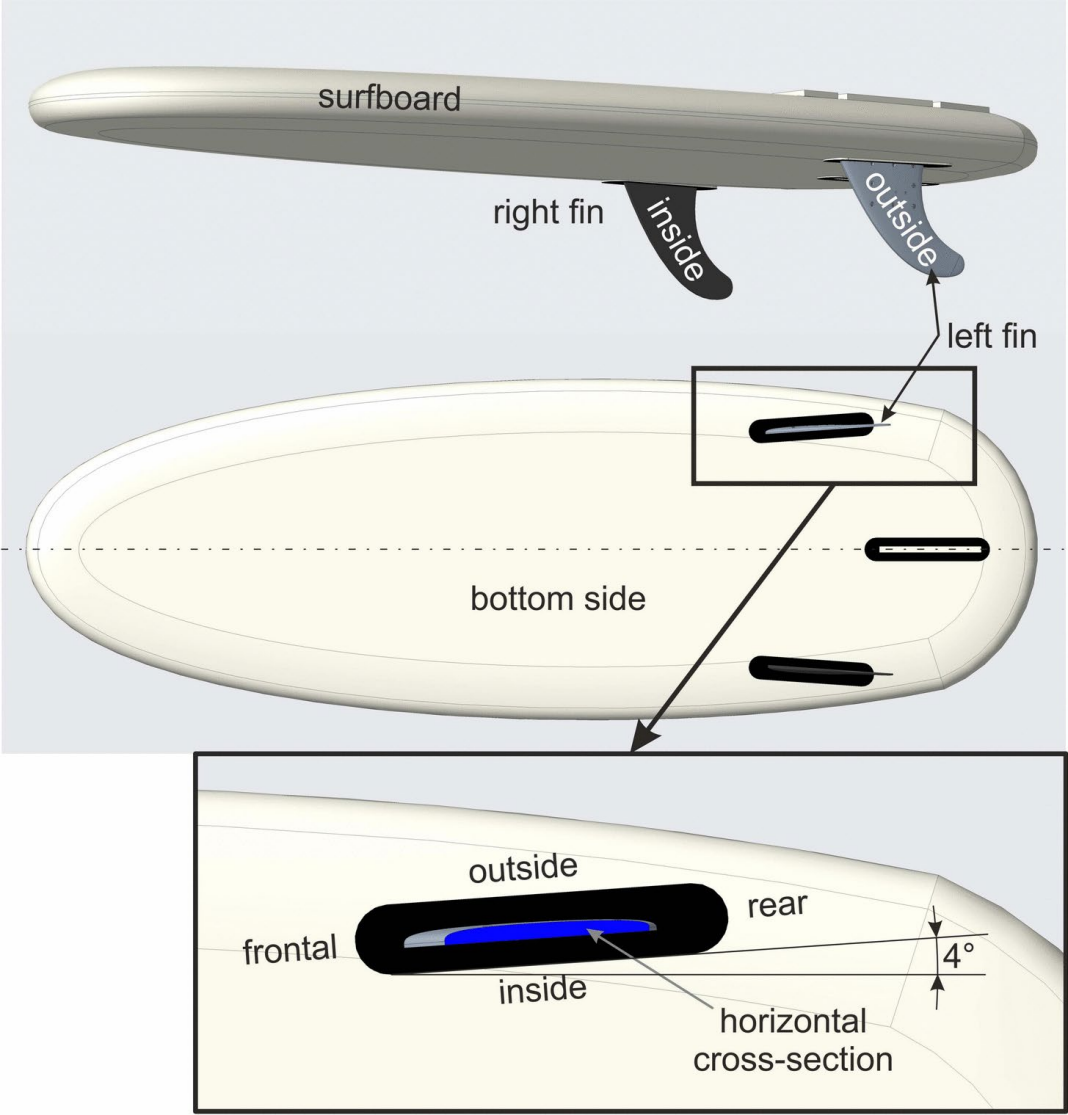
Surfboard configuration: Surfboard with a left and right fin omitting the central fin for better visibility (top), bottom view of the surfboard (middle) and close-up of the left equipped fin with the intersection surface colored in blue located at the sensor level and the toe in angle of $4^{\circ }$ (bottom).

The instrumented left fin of a classical 2-fin configuration was mounted in a Hypto Krypto FutureFlex (Haydenshapes Inc.) surfboard of size $5\,'4''\,\times \, 19\,1/2''\,\times \, 2\,''$ (length $\times$ width $\times$ thickness) and volume $26.24\,$l as displayed in Fig. 6. The fin was vertically tilted to produce a toe-in angle of $4^{\circ }$. In the bottom part of Fig. 6, the fin profile at the position of the four pressure sensors is colored in blue showing a wing profile with the curved surface indicated as “outside” facing the medium-lateral direction of the surfboard. The flat surface of the fin profile points to the in lateral-medium direction and is, therefore, indicated by “inside” as shown in the middle part of Fig. 6.

### Test setups and paradigm

#### Laboratory measurements in a water tank

**Fig. 7:**
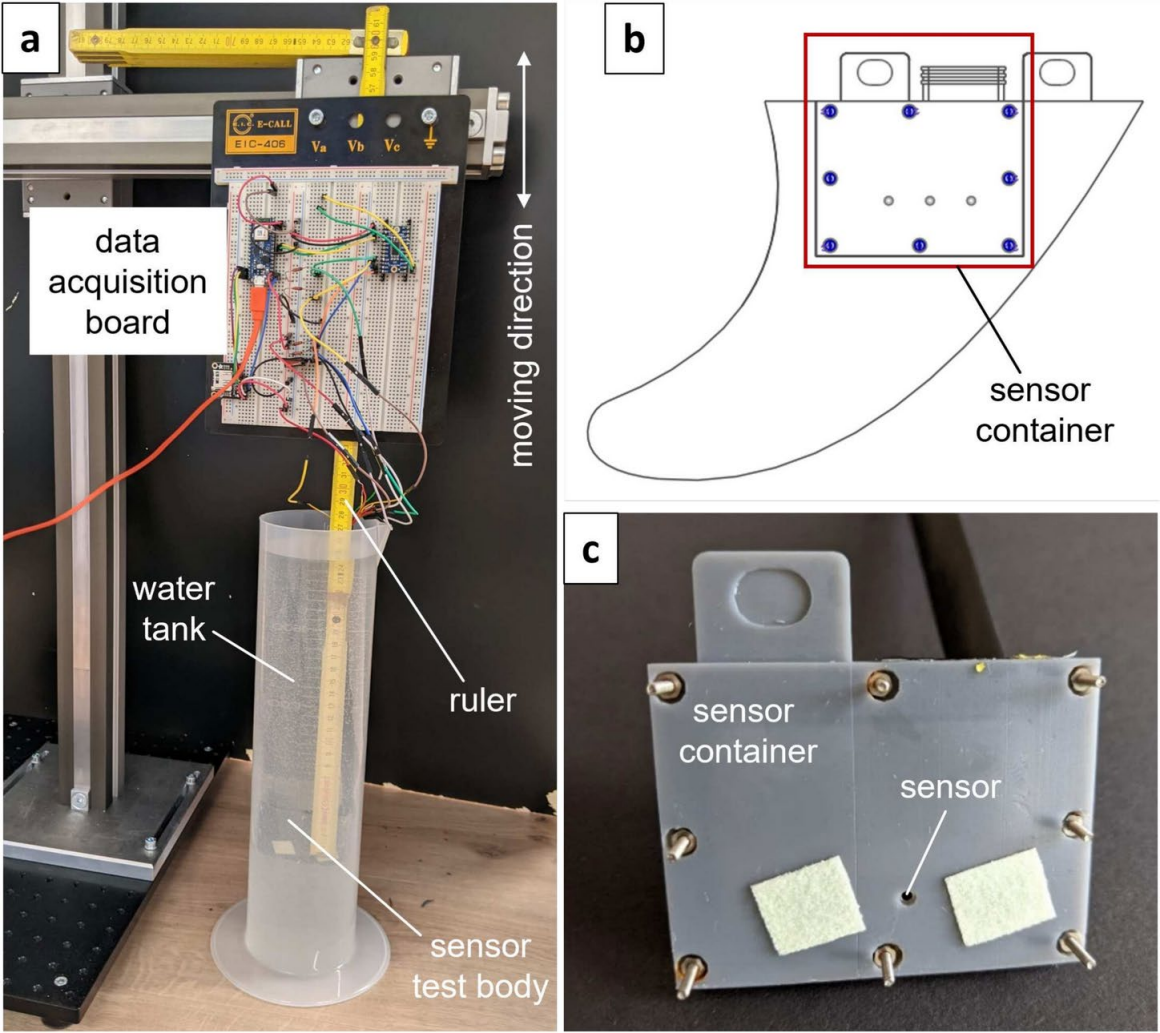
Experimental setup for the water tank measurements. (**a**) Photo of the test setup with the water tank, the folding ruler, the data acquisition board and the vertical traversing system. (**b**) Schematic of container including the pressure sensor in relation to the fin. (**c**) Photo of the sensor container.

The sensor and acquisition system was tested under laboratory condition using a transparent water tank depicted in Fig. 7a. Instead of the entire fin, a watertight container with two embedded pressure sensors was used as showing in Fig. 7b, c. The dimensions of the internal volume within the container was identical to the internal volume of the fin shown in the right portion of Fig. 5. The container was attached to the end of a folding ruler with the pressure sensors exactly positioned at $0\,$cm on the ruler scale. The ruler and the data acquisition board was mounted at a vertical traversing unit above the water tank to control the diving depth of the container. The depth could be read off from the folding ruler scale at water surface.

The depth of water was controlled in the range $L_{dw}\in [0\,\text {cm},\,25\,\text {cm}]$ corresponding to the hydrostatic pressure of $P_{dw}\in [0\,\text {Pa},\,2453\,\text {Pa}]$ relative to the atmospheric pressure. The measurements were performed at 11 constant levels of water depth, from $L_{dw}=0\,$cm to $L_{dw}=25\,$cm with a incremental step of $\Delta L_{wd}=2.5\,$cm. Thereby, the pressure was acquired sequentially from the two sensors with a sampling frequency for each sensor amounting $f_s=200\,$Hz with $1\,$ms delay between the measured values of the two sensors. At each depth of water, 600 samples were measured by each sensor.

#### Measurements during surfing on a river wave

The pressure measurements in surfing situations were carried out on the artificial river wave Fuchslochwelle in Nürnberg^36^ which was constructed within a bypass channel of *Pegnitz* river. A male surfer of advanced level ($43\,{\text{years}},\ 184\,{\text{cm}},\ 90\,$kg) performed several turns with the equipped surfboard on the standing river wave. During the surfing maneuvers, the surfer’s motion was simultaneously recorded with a GoPro Hero Black 5 camera (GoPro Inc., San Mateo, CA, USA) with a resolution of $1920\times 1080\,$pixels and using a frame rate of $f_f=30\,$Hz. The camera was positioned at central position upstream of the wave ramp mounted on a wooden beam that was laid across the inlet channel. At the time of the measurements the flow rate of the river was $6.2\,\text {m}^3/\text {s}$ producing a wave height of approximately $1\,$m. The corresponding flow velocity was estimated to be about $4\,$m/s.

The entire test procedure lasted $2:40\,$min subdivided in two parts. In the first part P1 lasting $2:10\,$min, the surfer just performed stationary surfing and small turns to get familiar with the instrumented surfboard. In the second part P2 which lasted approx. $30\,$s, the surfer performed 10 turns while crossing the entire channel five times.

Ethical clearance for field research was obtained from the Human Research Ethics Committee of the Friedrich-Alexander University Erlangen-Nürnberg under application number 23-312-ANF. Written consent was obtained from the male surfer.

## Data Availability

Data is provided within the manuscript. To request the data please send an email to the corresponding author.
